# Dry Eye Disease after Cataract Surgery: Study of its Determinants and Risk Factors

**DOI:** 10.4274/tjo.galenos.2019.45538

**Published:** 2020-06-27

**Authors:** Pragati Garg, Aditi Gupta, Nishi Tandon, Priyanka Raj

**Affiliations:** 1 Era’s Lucknow Medical College and Hospital, Clinic of Ophthalmology, Lucknow, India; 2 Era’s Lucknow Medical College and Hospital, Clinic of Pathology, Lucknow, India

**Keywords:** Cataract, dry eye disease, Schirmer’s test, Ocular Surface Disease Index, phacoemulsification

## Abstract

**Objectives:**

To study the incidence of dry eye and its determinants in patients undergoing cataract surgery.

**Materials and Methods:**

One hundred twenty patients with senile cataract underwent Schirmer’s test, tear break-up time (TBUT) test, lissamine green staining of the cornea and conjunctiva, and Ocular Surface Disease Index (OSDI) for evaluation of dry eye preoperatively and again at first and second follow-up examinations at 1 week and 1 month after cataract surgery.

**Results:**

Mean age of the patients was 59.25+9.77 years and 73 (60.8%) were men. None of the patients had dry eye at the time of enrollment as per the criteria of our study. Postoperatively, Schirmer’s test values ranged from 12-35 mm and 8-24 mm at first and second follow-ups, respectively. Mean TBUT was 13.16±2.45 and 9.64±2.20 seconds, while lissamine green staining score was 3 in 67 (55.8%) and 1 in 67 (55.8%) subjects at first and second follow-up, respectively. OSDI values ranged from 1-30 and 10-33 with a mean of 25.97±5.34 and 11.96±7.47 respectively at first and second follow-up. At first follow-up, 89.1% of the 56 patients who underwent phacoemulsification were found to have grade 2 dry eye (p<0.001), while 92.2% of the 64 patients who underwent small-incision cataract surgery (SICS) had grade 2 dry eye (p<0.001). At second follow-up, grade 0 dry eye was observed in 92.2% of the patients who underwent phacoemulsification and 82.1% of the patients who underwent SICS (p<0.001).

**Conclusion:**

The incidence of dry eye after cataract surgery was high and mostly independent of demographic and anthropometric profile, type of surgical procedure, time of microscope exposure, and amount of energy used. This dryness was transient in nature and showed a declining trend, tending to achieve normalization by the end of 1 month.

## Introduction

Dry eye disease (DED) is defined as “a disorder of the tear film due to reduced tear production or excessive tear evaporation, which causes damage to the inter-palpebral ocular surface and is associated with symptoms of ocular discomfort and/or visual symptoms”.^1^ A more descriptive definition given by the dry eye workshop defines it as “a multifactorial disease of the tear film and ocular surface that results in symptoms of discomfort, visual disturbance, and tear film instability with potential damage to the ocular surface. It is accompanied by increased osmolality of the tear film and inflammation of the ocular surface”.^2^

The etiology of dry eye syndrome has been attributed to a number of causes and factors that include old age, gender, disorders affecting the connective tissue, metabolic disorders like diabetes and hypertension, contact lens usage, drugs like antihistamines, anticholinergics, antidepressants, oral contraceptives and topical eye drops containing preservatives, and ocular diseases like blepharitis, chronic conjunctivitis, meibomitis, and pterygium.^3,4,5^

Apart from the conventional risk factors of dry eye syndrome, it has been seen that some surgical procedures related to the anterior segment like photorefractive keratectomy, laser-assisted in situ keratomileusis, and cataract surgery are also responsible for causing dry eye syndrome or aggravating existing symptoms of dry eye.^6,7,8^ Surgical procedures like cataract surgery cause denervation of the cornea, which results in impaired epithelial wound healing, increased epithelial permeability, decreased epithelial metabolic activity, and loss of cytoskeletal structures associated with cellular adhesion. The incidence of dry eye syndrome among patients undergoing cataract surgery has been shown to be dependent on a host of factors including type of procedure, type of ophthalmic solution being used^9^, intraoperative medication^10^, coexistent systemic disorders^11^, operating microscope light exposure and cumulative dissipated energy (CDE) used during the procedure^12^, and time since surgery.^13^

Considering the fact that occurrence of dry eye syndrome after cataract surgery could be dependent on a number of factors like the type of surgery^9^, intraoperative exposure^12^, and energy used during phacoemulsification^12^, it is essential that a proper risk assessment be done in both phacoemulsification as well as small-incision cataract surgery (SICS) procedures. Thus, the present study was carried out with an aim to assess the incidence of dry eye syndrome and its determinants among patients undergoing cataract surgery at a tertiary care center in North India.

## Materials and Methods

This hospital-based observational study was carried out in the Clinic of Ophthalmology of Era’s Lucknow Medical College and Hospital, Lucknow, a tertiary care-center in North India, over a period of 18 months. We initially enrolled 176 subjects with senile cataract and without pre-existing dry eye syndrome. Subjects underwent detailed history and ocular examination and those with ocular conditions that can contribute to the occurrence of dry eye, such as lid disorders (blepharitis, ectropion, entropion), contact lens wear, allergic conjunctivitis, any past ocular surgeries, chronic conjunctivitis, exposure keratitis, contact dermatitis, and Bell’s palsy; those with systemic conditions like diabetes mellitus, hypertension^14^, thyroid-associated diseases, lupus, rheumatoid arthritis, scleroderma, Sjögren’s syndrome, vitamin A deficiency, and other factors like smoking^15^; and those with continuous long-term use of ocular or systemic medications (antihistaminics, antidepressants, decongestants, beta blocker drugs, diuretics, and aspirin) were excluded from this study. After excluding subjects who failed to meet the inclusion and exclusion criteria, did not give consent, or were lost to follow-up, there were 120 patients.

All the subjects included in the study best corrected visual acuity was assessed by Snellen chart and intraocular pressure by Goldmann applanation tonometer. Detailed slit-lamp examination was done and the fundus was examined by indirect ophthalmoscopy.

Schirmer’s test, tear break-up time (TBUT) test, lissamine green staining of the cornea and conjunctiva, and Ocular Surface Disease Index (OSDI) were carried out for evaluation of dry eye.

Schirmer’s test was done to test basal and reflex tear secretion using a specialized Schirmer’s strip prepared from Whatman filter paper no. 41 measuring 40´5 mm, marked 0 to 35 mm. Depending on the wetting of the strip, the results of Schirmer’s test were graded as: >10 mm, normal (grade 0); 5-10 mm, mild (grade 1); 3-4 mm, moderate (grade 2); 0-2 mm, severe (grade 3).^16^

TBUT was assessed to test tear film stability and meibomian gland disorder and the grading was done depending upon the time between the last blink and the appearance of a dry spot. TBUT less than 10 s was abnormal and graded as: >10 s, normal (grade 0); 3.1-6 s, moderate (grade 2); 6.1-10 s, fair (grade 1); <3 s, poor (grade 3).^16^

Lissamine green staining of the ocular surface was done to assess the dead and devitalized cells on the ocular surface. Results were graded as: 0, no dry eye; 1, mild dry eye; 2, moderate dry eye; and 3, severe dry eye. OSDI is a 12-item evaluation for dry eye assessed on a scale of 0 to 100, with higher scores representing greater disability. The index demonstrates sensitivity and specificity in distinguishing between normal subjects and patients with dry eye syndrome. The criteria used for the grading was: 0-12, normal; 13-22, mild; 23-32, moderate; and 33-100, severe.^17,18^

Risk factors such as pre-anesthetic medication, shape of incision, type of cataract surgery (phacoemulsification/SICS), microscope light exposure, CDE manipulation of ocular surface tissue, and intra- and postoperative medications were taken into consideration. Of these factors, all cases had the same pre-anesthetic medication, shape of incision, intra- and postoperative medications (which included a combination of antibiotic and steroid, non-steroidal anti-inflammatory and intraocular pressure-lowering topical eye drops from the same pharmaceutical brands, instilled at similar frequencies), and operating surgeon.

The patients were followed up 1 week and 1 month after the surgery. Evaluations of all dry eye parameters were repeated on both occasions.

The study was conducted after ethical approval by the institutional ethics committee in accordance with international agreements and the Declaration of Helsinki, and informed and written consent was obtained from all the subjects included in the study.

### Statistical Analysis

The statistical analysis was done using Statistical Package for Social Sciences version 21.0 statistical software. The values were presented in number (%) and mean ± standard deviation. P values of <0.05 were considered significant and <0.001 as highly significant.

## Results

Out of 120 subjects evaluated, the largest age group was 61-70 years (n=43, 35.83%), followed by those aged 51-60 years (n=40, 33.33%), <50 years (n=30, 25%), and >70 years (n=7, 5.83%). The mean age of the patients was 59.25+9.77 years and most were men (n=73, 60.83%). The male to female ratio was 1.55:1.

Most of the patients were from rural areas (n=103, 85.83%); subjects from urban areas comprised only 14.17% (n=17) of the study population. With respect to occupation, the largest group was homemakers (n=42, 35%), followed by farmers (n=33, 27.5%), skilled laborers (n=17, 14.17%), teachers (n=16, 13.33%), and shopkeepers (n=12, 10%). Using body mass index (BMI) criteria, the nutritional status of 114 patients (95%) was adjudged as normal weight (18.5-25.0 kg/m^2^) and the other 6 (5%) fell in the overweight category (25.1-30 kg/m^2^). None of the patients evaluated were underweight (<18.5 kg/m^2^) or obese (>30 kg/m^2^) (Table 1).

The most common ocular symptoms of the patients were photophobia (55.83%), itching (50.83%), watering (45.83%), burning sensation (45%), eye pain (41.67%), redness of eyes (39.17%), lid heaviness (36.67%), foreign body sensation (28.33%) and discharge (27.50%) (Table 2).

In preoperative clinical assessment of dry eye, Schirmer’s test values ranged from 15 to 35 mm with a mean of 27.23±4.38 mm. Mean TBUT was 13.50±1.89 (range 10-18 s). Lissamine green staining score was 1 in 79 (65.8%) and 2 in 41 (34.2%) cases.

OSDI values ranged from 1 to 12 with a mean of 6.48+2.61. None of the patients had dry eye at the time of enrollment, as per the inclusion criteria of the study (Table 3).

Postoperative Schirmer’s test values ranged from 12 to 35 mm at 1 week and 8 to 24 mm at 1 month. Mean TBUT was 13.16±2.45 and 9.64±2.20 s, while lissamine green staining score was 3 in 67 (55.8%) and 1 in 67 (55.8%) subjects at 1 week and 1 month follow-up, respectively. OSDI values ranged from 1 to 30 (mean 25.97±5.34) at 1 week and 10 to 33 (mean 11.96±7.47) at 1 month (Table 3).

Cataract surgery was performed using the phacoemulsification technique in 53.33% of patients while remaining 46.67% underwent SICS. The incidence of dry eye was 89.1% and 15.6% in the phacoemulsification group at 1 week and 1 month, compared to 92.9% and 26.8% in the SICS group at the corresponding time points. Although the incidence of dry eye was higher after SICS as compared to phacoemulsification at both time points, the differences were not significant statistically (p>0.05) (Table 4).

We compared OSDI grade among the subjects preoperatively and postoperatively. On evaluating all the subjects together, irrespective of the technique of cataract surgery, all of the 120 subjects (100%) were in grade 0 preoperatively, while at postoperative 1 week, only 8 patients (6.7%) had grade 0, 3 patients (2.5%) had grade 1, and 109 (90.8%) had grade 2 dry eye. At 1 month follow-up, 105 patients (87.5) had grade 0, 14 (11.7%) had grade 1, and only 1 (0.8%) had grade 2 dry eye according to the OSDI scale.

When comparing OSDI grade among the patients according to cataract surgery technique, all 64 (100%) of the patients who underwent phacoemulsification and 56 (100%) patients who underwent SICS had grade 0 dry eye preoperatively. At postoperative 1 week, 89.1% of the phacoemulsification group had grade 2 dry eye (p<0.001) and 92.9% of the SICS group had grade 2 dry eye (p<0.001). At 1-month follow-up, 92.2% of the phacoemulsification group had grade 0 and the other 7.8% had grade 1 dry eye. None of the patients had grade 3 dry eye at 1 month and the results were statistically significant (p<0.001). Of the 56 patients who underwent SICS, 82.1% had grade 0, 16.1% had grade 1, and only 1.8% had grade 2 dry eye at postoperative 1 month and the results were statistically significant (p<0.001) (Table 5).

On overall evaluation, the majority of patients (n=65, 54.2%) had microscope exposure time within 10-15 minimum (min), followed by 16-20 min (n=34, 28.3%), 21-25 min (n=11, 9.2%), and 26-30 min (n=10, 8.3%). In the phacoemulsification group, most patients (n=53, 82.8%) had microscope exposure time of 10-15 min, followed by 16-20 min (n=10, 15.6%), and 21-25 min (n=1, 1.6%). However, in the SICS group, the most frequent microscope exposure time was 16-20 min (n=24, 42.9%), followed by 10-15 min (n=12, 21.4%), and 21-25 and 26-30 min (n=10, 17.9% each).

On overall evaluation as well as among patients undergoing SICS, microscope exposure time did not show a significant association with the incidence of dry eye at postoperative 1 week or 1 month. However, in the phacoemulsification group, exposure time >15 min was found to be significantly associated with an increased risk of dry eye at 1 week (p=0.009) (Table 6).

Among phacoemulsification cases, the majority (n=33, 51.6%) underwent the procedure with 8.0-11.5% CDE, followed by 11.6-15.5% (37.5%) and 15.6-19.0% CDE (10.9%). On evaluating the relationship between CDE and incidence of dry eye at 1 week and 1 month, the association was not found to be significant (Table 7).

On looking for the pattern of change in values of different dry eye parameters from baseline at 1 week and 1 month, we observed a significant decline in mean Schirmer’s test and TBUT values at 1 week compared to baseline; however, by 1 month the change from baseline was not statistically significant. In LG staining, the proportion of those having scores of 3 and 4 was significantly higher at 1 week but returned to baseline by 1 month.

Mean OSDI values were 6.48±2.61 at baseline and reached 25.98±5.34 at 1 week and 10.53±4.99 at 1 month. The change from baseline was statistically significant at both follow-ups. Compared to baseline, when all patients had OSDI grade 0, the proportion of patients having OSDI grades 2 and 3 was significantly higher at 1 week and the proportion having grades 1 and 2 was significantly higher at 1 month (Table 8).

## Discussion

Cataract surgery is perhaps one of the most frequently performed ophthalmic procedures.^19^ However, like any other surgery, it is not free from post-operative complications. The most common complications are postoperative inflammatory reaction, increase in intraocular pressure, cystoid macular edema, and significant post-operative astigmatism. Along with these complications, the patients also complain of dry eye symptoms of grittiness, foreign body sensation, and burning sensation, which are commonly overlooked.

Dry eye following cataract surgery has been shown to have a high variability in its incidence, ranging from 9.8 to 96.6.^20,21,22,23,24,25,26,27,28,29,30,31,32,33,34,35,36,37,38,39,40,41,42,43,44,45,46,47,48,49,50,51,52,53,54,55,56,57,58,59,60,61,62,63,64,65^ The incidence of dry eye among patients undergoing cataract surgery has been shown to be dependent on a host of factors including the type of procedure (SICS/phacoemulsification), type of ophthalmic solution used, intra- and postoperative medications, coexistent systemic disorders, operating microscope exposure time, CDE during phacoemulsification, and time since surgery. But none of them have been confirmed to be individually responsible for causing dry eye.

Hence to study the incidence of dry eye and its determinants in patients undergoing cataract surgery we enrolled 120 patients who were scheduled to undergo unilateral cataract surgery. The mean age of the patients was 59.25±9.77 years, a finding consistent with a study conducted by Dodia et al.^59^ in which the mean age of the patients was 60.35 years. However, the majority of patients in our study were males (60.83%), in contrast to previous studies in which the majority of patients were females.^57,13,60,62^ This is probably due to estrogen changes related with menopause which results in higher risk of age-related cataract among females as compared to males.^66,67^ With respect to domicile and occupational profile of patients, most studies did not report on this aspect. In our study, homemakers (35%) and farmers (27.5%) comprised the most dominant occupational groups. We feel that considering differences in sanitary conditions and environment, place of residence and occupation could have a role in affecting the dry eye prevalence at 1-month follow-up, when patients have resumed their activities of daily living.^68,69,70^

Nearly all of the patients in our study had BMI in normal range (18.5-24.9 kg/m^2^) (95%) and remaining 5% of patients were in the overweight category (BMI 25-29.9 kg/m^2^). Although no significant associations have been reported between DED and BMI in past, this patient characteristic was included in our assessment as BMI is known to have association with various systemic illnesses, which in turn might have a contributing role in the development of dry eye following cataract surgery.

Preoperatively, the majority of patients had complaints of photophobia (55.83%) and itching (50.83%). On average, each patient had 3.7 symptoms from amongst foreign body sensation, burning sensation, discharge from eye, itching, lid heaviness, redness of eye, photophobia, watering, and eye pain. None of the previous studies reports any assessment of preoperative symptoms. These symptoms were recorded and analyzed using OSDI grading, which showed all of them to be within the normal limits of dry eye severity grading preoperatively. This was corroborated, as all the patients had dry eye parameters (Schirmer’s test, TBUT, lissamine green staining, and OSDI) within the normal range when these investigations were conducted.

In our study, both phacoemulsification and SICS procedures were taken into account in order to assess the impact of the type of surgery on dry eye incidence. Phacoemulsification accounted for 53.33% and SICS 46.67% of the operations in our study. Few studies have included both SICS and phacoemulsification cases.^60,61,71^

At 1 week after surgery, Schirmer’s test, TBUT, and OSDI findings suggestive of dry eye were seen in 87.5%, 69.2%, and 91.7% of the patients, respectively. For the purposes of the present study, we accepted OSDI-evaluated dry eye as representing dry eye. Thus, the prevalence of dry eye was 91.7% in the present study. The reported prevalence of dry eye varies considerably in different studies, which might be dependent on various determinants as well as methods of evaluating dry eye. In the present study, we could see that while TBUT criteria detected dry eye in 69.2% of the patients, OSDI resulted in 91.7% prevalence of dry eye, thus increasing the prevalence by 1.33 times.

A considerable difference in the incidence of dry eye can be seen in different studies. Kasetsuwan et al.^20^ conducted their study that followed up patients at days 0, 7, 30 and 90, and reported that the severity of dry eye peaked at postoperative 7 days. Venugopal et al.^58^ on the other hand, evaluated data for patients from postoperative weeks 2 through 6 in 58.8% of their study population and from 6 weeks to 2 years in remaining 41.2% of their patients. Dodia et al.^59^ evaluated dry eye incidence at postoperative day 1, 7, and 45 and reported the peak incidence at day 1. Interestingly, most of the studies did not conduct any preoperative assessment for dry eye, and hence it was difficult to assess whether the dry eye incidence reported in the study was a continuation of preexisting dry eye syndrome or was a response to cataract surgery. In their study following patients for up to 2 years after cataract surgery, Venugopal et al.^58^ did not report the environmental and occupational risk and evaluated dry eye incidence as an outcome of cataract surgery without having any baseline data. In another study, Cetinkaya et al.^64^ made assessments for up to 2 years but had neither preoperative data nor data related to environmental and occupational risk factors. Moreover, most previous studies did not assess symptomatic risk factors for dry eye. The relatively higher burden of dry eye incidence in our study could be due to the fact that the study population had more than 3 symptomatic risk factors on average even before cataract surgery, and they could have influenced the incidence of dry eye since it is one of the most common comorbid conditions associated with cataract.^24^

In the present study, we also made a detailed assessment of different risk factors and then evaluated their impact on dry eye incidence, but we failed to find any association between age, gender, place of residence, occupation, BMI, or type of surgery (SICS/phacoemulsification) and the incidence of dry eye at postoperative 7 days and 1 month, similar to Venugopal et al.^58^

In the present study, we also evaluated the effect of microscope exposure time on dry eye incidence. We determined that overall and in patients undergoing SICS, microscope exposure time was not significantly associated with the incidence of dry eye at 1-week or 1-month follow-up. However, in the phacoemulsification group, exposure time >15 min was found to be significantly associated with an increased risk of dry eye at first follow-up. Prolonged microscope light exposure time has been correlated with reduced TBUT and temporarily worsened symptoms.^33^ In the present study, we did not find a significant association between CDE and incidence of dry eye at 1 week or 1 month among patients undergoing phacoemulsification. This is consistent with observations made by Sahu et al.,^13^ Rizvi et al.,^61^ and Sengupta and Banerji^68^ who also detected no significant difference between the phacoemulsification and SICS groups, similar to the findings of present study. Yu et al.^63^ also reported that type of surgery did not have an impact on dry eye incidence.

In the present study, nearly all the dry eye tests indicated mild dry eye (for OSDI and Schirmer’s: 85.8% mild, TBUT: 65% moderate). Similarly, most previous studies also reported a predominance of mild dry eye (53.32% by Venugopal^58^ and 58.06% by Manjula et al.^73^). However, Jayashree et al.^72^ reported a predominance of severe dry eye.

At postoperative 1 month, the incidence of dry eye as per Schirmer’s test, TBUT, and OSDI was 16.7%, 19.2% and 20.8%, respectively. Compared to 1-week follow-up, there was a significant change at 1 month. As far as pattern of change in different dry eye parameters was concerned, we observed that mean Schirmer’s test result was 27.22±4.40 preoperatively and 12.91±2.95 and 24.61±6.32 mm at postoperative 1 week and 1 month, respectively. At the same time points, mean TBUT was 13.50±1.89, 9.64±2.20, and 13.16±2.45 seconds, respectively, and the proportion of patients with lissamine green staining scores of 2 or higher was 34.2%, 97.5%, and 44.2%, respectively. In general, as compared to preoperative assessment, all of the values peaked at 1 week and then tended to decline by the end of 1 month. However, none of these parameters returned to baseline even at 1-month follow-up. These findings indicate that the peak impact of cataract surgery in terms of dry eye was during the first week and tended to decline thereafter.

Similar to our findings, Kasetsuwan et al.^20^ also observed that severity of dry eye peaked 7 days after cataract surgery as measured by OSDI questionnaire and clinical tests which showed rapid and gradual improvements within 1 and 3 months post-surgery. This was similar to observations made by Sahu et al.,^13^ who found a deterioration in the Schirmer’s test I, tear meniscus height, TBUT, and lissamine green staining of the cornea and conjunctiva following phacoemulsification surgery which started improving after 1 month. They reported that despite the improvements observed at 1 month, as observed in the present study, preoperative values were not achieved until 2 months after surgery. The majority of researchers observe the impact of cataract surgery to be a transient one and recoverable within 3 to 6 months.^59,60,61,64,71^

The findings of the present study indicated that there is a high incidence of dry eye following cataract surgery. This may be due to corneal nerve transection, which results in impaired epithelial wound healing, increased permeability, decreased epithelial metabolic activity, and loss of cytoskeletal structures leading to decreased corneal sensitivity with subsequent reduction in tear production, as found by Sutu et al.^25^ Elevation of inflammatory response leading to recruitment of neutrophils and macrophages and production of free radicals, proteolytic enzymes, and cyclooxygenase is also considered to be a key factor in the development of dry eye. Topical anesthetics and pre- and postoperative preservative-containing eye drops also contribute to the inflammatory reaction.

## Conclusion

The findings of the present study showed that incidence of dry eye after cataract surgery was quite high, irrespective and nearly independent of variables such as demographic and anthropometric profile, type of surgical procedure, microscope exposure time and amount of energy used. The trends showed that this dryness was transient in nature and tended toward normalization at the end of 1 month. Due to the limited follow-up time of the study, we could not ascertain when all the patients returned to normal. Further studies with longer duration of follow-up targeted to assess time taken to attain normal status are recommended. Moreover, long-term residual dry eye following cataract surgery also needs to be investigated.

Thus, dry eye is seen to be a common complaint post-cataract surgery that significantly affects patient satisfaction despite excellent visual recovery and needs to be addressed. In light of the high incidence of dry eye following cataract surgery, we recommend the use of an appropriate lubricating agent during postoperative recovery for 2 to 3 months after cataract surgery in order to avoid dry eye-related complications following surgery and provide symptomatic relief to patients.

## Figures and Tables

**Table 1:**
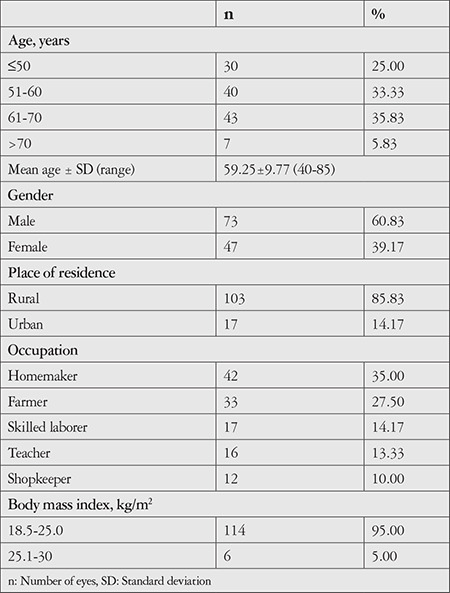
Demographic profile of the patients (n=120)

**Table 2:**
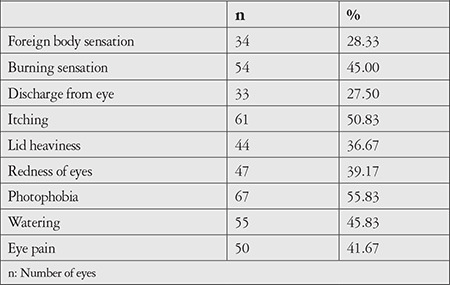
Presenting symptoms of included patients (n=120)

**Table 3:**
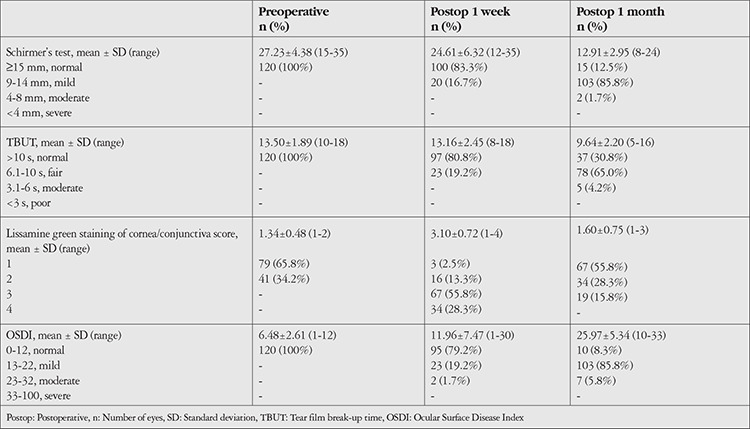
Clinical assessment (for dry eye-related tests) preoperatively and at postoperative 1 week and 1 month

**Table 4:**
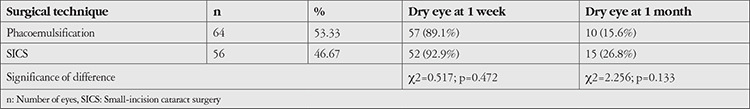
Comparison of dry eye incidence at postoperative 1 week and 1 month between phacoemulsification and smallincision cataract surgery groups

**Table 5:**
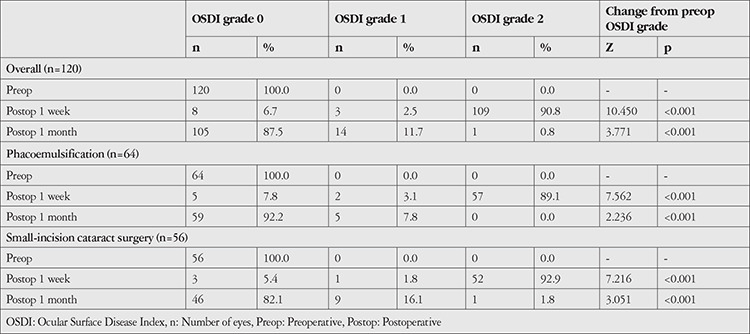
Comparison of Ocular Surface Disease Index grade preoperatively and at postoperative 1 week and 1 month (Wilcoxon Signed Rank test)

**Table 6:**
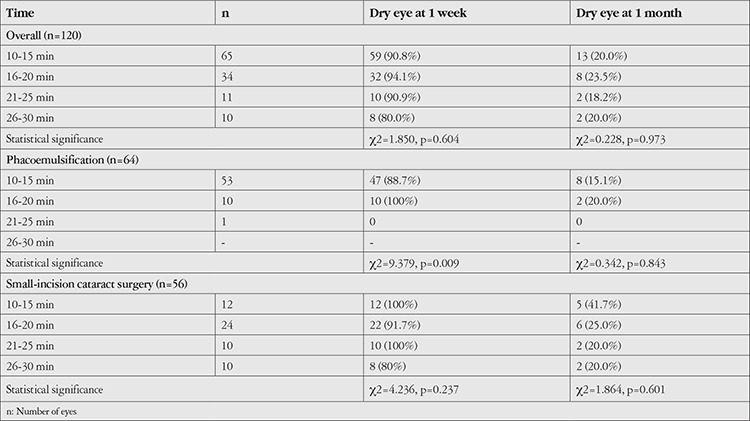
Association of microscope exposure time with dry eye at 1 week and 1 month after cataract surgery

**Table 7:**
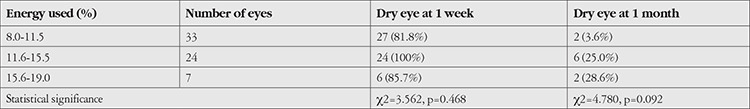
Relationship between cumulative dissipated energy (%) and dry eye (only phacoemulsification cases, n=64)

**Table 8:**
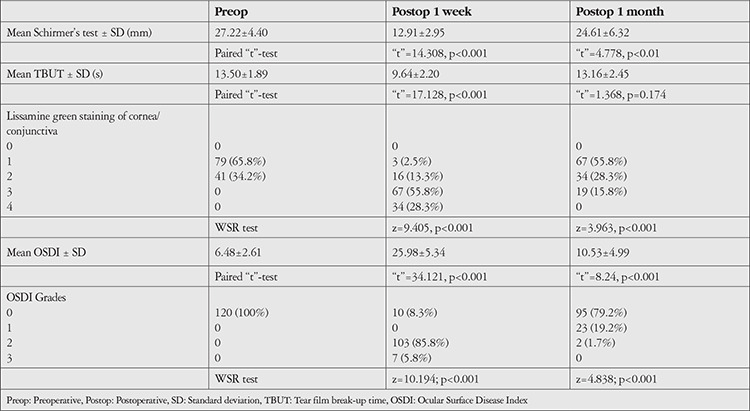
Changes from baseline (preoperative values) in different clinical parameters at postoperative 1 week and 1 month (n=120)
